# Implementation of a salt substitute intervention using social marketing in resourced-limited communities in Peru: a process evaluation study

**DOI:** 10.3389/fpubh.2023.1068624

**Published:** 2023-05-19

**Authors:** María Lazo-Porras, Adela Del Valle, David Beran, Maria Amalia Pesantes, Silvana Perez-Leon, Vilarmina Ponce-Lucero, Antonio Bernabe-Ortiz, María Kathia Cárdenas, François Chappuis, Pablo Perel, J. Jaime Miranda, Francisco Diez-Canseco

**Affiliations:** ^1^CRONICAS Center of Excellence in Chronic Diseases, Universidad Peruana Cayetano Heredia, Lima, Peru; ^2^Division of Tropical and Humanitarian Medicine, University of Geneva and Geneva University Hospitals, Geneva, Switzerland; ^3^Dickinson College, Department of Anthropology, Carlisle, PA, United States; ^4^Centre for Global Chronic Conditions, London School of Hygiene and Tropical Medicine, London, United Kingdom; ^5^School of Medicine, Universidad Peruana Cayetano Heredia, Lima, Peru

**Keywords:** process evaluation, salt replacement, context, acceptability, fidelity, low- and middle-income countries, hypertension

## Abstract

**Objective:**

This study aimed to conduct a process evaluation of a salt substitute trial conducted in Peru.

**Methods:**

Through semi-structured interviews of intervention participants, we documented and analyzed process evaluation variables as defined by the Medical Research Council Framework. This study was a stepped wedge trial conducted in Tumbes, Peru in 2014. The intervention was a community-wide replacement of regular salt (100% sodium) with “Salt Liz” (75% sodium and 25% potassium) using social marketing strategies to promote the adoption and continued use of the salt substitute in daily life. The components of the social marketing campaign included entertainment educational activities and local product promoters (“*Amigas de Liz*”). Another component of the intervention was the Salt Liz spoon to help guide the amount of salt that families should consume. The process evaluation variables measured were the context, mechanism of action, and implementation outcomes (acceptability, fidelity and adoption, perceptions, and feedback).

**Results:**

In total, 60 women were interviewed, 20 with hypertension and 40 without hypertension. Regarding context, common characteristics across the four villages included residents who primarily ate their meals at home and women who were responsible for household food preparation. As the mechanism of action, most participants did not notice a difference in the flavor between regular salt and Salt Liz; those that did notice a difference took around 2 weeks to become accustomed to the taste of the salt substitute. In terms of implementation outcomes, the Salt Liz was accepted by villagers and factors explaining this acceptability included that it was perceived as a “high quality” salt and as having a positive effect on one's health. Participants recognized that the Salt Liz is healthier than regular salt and that it can help prevent or control hypertension. However, most participants could not accurately recall how the compositions of the Salt Liz and regular salt differed and the role they play in hypertension. Although the use of the Salt Liz was far-reaching at the community level, the use of the Salt Liz spoon was poor. Educational entertainment activities were well-received, and most participants enjoyed them despite not always being active participants but rather sideline observers.

**Conclusion:**

This process evaluation identifies key intervention components that enabled a successful trial. Seeking and incorporating feedback from the target population helps deepen the understanding of contextual factors that influence an intervention's success. Furthermore, feedback received can aid the development of the intervention product. Some factors that can be improved for future interventions are acknowledged.

**Clinical trial registration:**

NCT01960972.

## 1. Introduction

An estimated 1.28 billion adults aged 30–79 years worldwide have hypertension, two-thirds living in low- and middle-income countries (LMIC) (1), where the prevalence of hypertension is 17% in this age group (2). The World Health Organization (WHO) has outlined various strategies for preventing or managing hypertension, including the reduction of dietary sodium intake (3, 4). Lowering the consumption of sodium is a cost-effective way for improving hypertension and cardiovascular health outcomes (4). One strategy for reducing sodium intake is replacing regular salt (100% sodium chloride, NaCl) with a salt substitute made up of a combination of NaCl and KCl (potassium chloride). Studies have found that salt substitution strategies improve blood pressure levels in people with hypertension (5, 6) and cardiovascular outcomes including mortality (7).

A stepped wedge trial (SALT project NCT01960972) was conducted in six villages in the region of Tumbes, Peru, between 2014 and 2017 (8). In the trial, the regular salt consumed by intervention villages was replaced with a salt substitute (branded as “Salt Liz”) embedded within a social marketing campaign to encourage its uptake and continued use. The aim of the trial was to estimate the effect of the intervention on community-wide blood pressure levels and hypertension prevention. The project's findings showed that the overall intervened population had an average reduction of 1.29 mmHg in systolic and 0.76 mmHg in diastolic blood pressure. Likewise, the use of the salt substitute was associated with a 51% reduced risk of developing hypertension compared to the control [95% confidence interval (29%−66%)]. Only a random subgroup of participants (*n* = 600) provided urine samples for analysis both at the start and the end of the intervention. The study did not find a difference in the levels of sodium measured at both moments; however, the higher levels of potassium at the end of the trial were found compared to the baseline, which supports that the Salt Liz was consumed by the population (8, 9).

Despite the project's successful impact on hypertension-related health outcomes, the trial results do not reveal what intervention components worked well, what challenges were faced during the implementation, and what improvements need to be considered for future scale-up (9–11). We conducted a process evaluation to (i) identify contextual factors that played a role in the implementation of the salt substitute; (ii) recognize the mechanisms of action by which participants incorporated the salt substitute in daily life; (iii) describe the acceptability of the salt substitute and identify the factors that influenced it; (iv) evaluate the fidelity of the intervention components; (v) explore participant perceptions surrounding social marketing campaign components used to promote the salt substitute; and (vi) gather feedback to improve the future interventions and inform future scale-up efforts.

## 2. Methods

### 2.1. Setting

A stepped wedge trial (SALT project NCT01960972) was conducted in six villages in Tumbes, a coastal region in northern Peru bordering Ecuador. The prevalence and incidence rates of hypertension in Tumbes are 12.4% and 9.88 per 100 person-years, respectively (13), estimates that are above the national averages (14). Six out of more than 100 semi-urban villages were randomly selected for the intervention. Out of these six intervention villages, we collected data from four to inform this process evaluation. More detailed information about the setting is available elsewhere (8).

### 2.2. Preliminary phase of the SALT project

The SALT project had a preliminary phase that included a qualitative formative study. The intervention that was tested during the trial was developed and piloted during the preliminary phase.

#### 2.2.1. Formative phase

**Figure 1:**
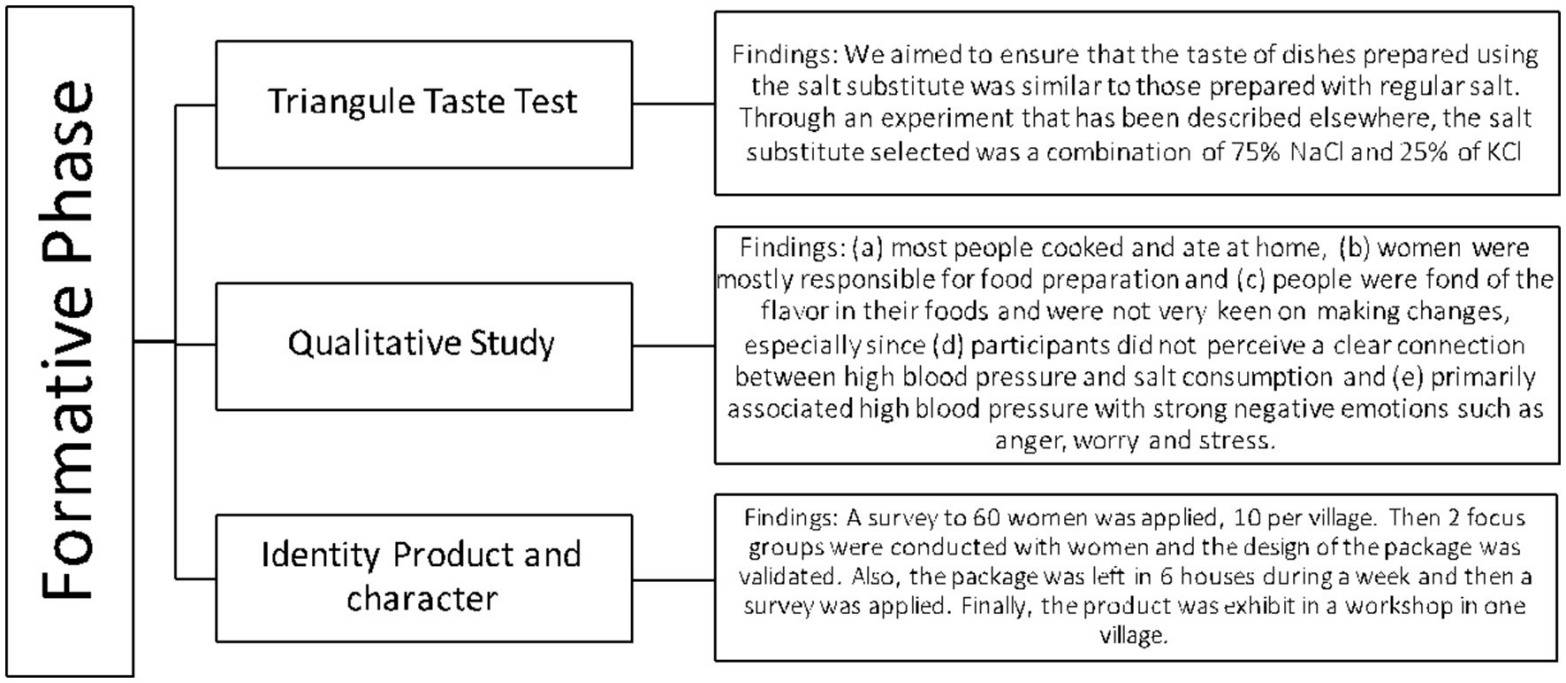
The formative phase of the SALT study.

To collect relevant information for designing the intervention, three formative studies were conducted. The first study helped identify the ideal proportions of sodium and potassium in the substitute that would not significantly alter the taste of the food (15). The second, a qualitative study, informed about the development of the social marketing campaign (16). Finally, the third helped define product branding and identity for the salt substitute. More information is available in Figure 1.

#### 2.2.2. Intervention

This study involved a complex intervention that (i) retrieved and replaced, free of charge, the regular salt with Salt Liz in enrolled households and small businesses and (ii) launched a campaign using social marketing, targeting women villagers who were responsible for household food preparation. In countries like Peru, with a diversity of culinary flavors ranging from salty to spicy, it can prove challenging to change predispositions toward saltier foods in favor of a reduced-sodium diet. Social marketing's use of “*marketing concepts with other approaches to influence behaviors that benefit individuals and communities”* (17) was seen as a fitting tool for introducing a new product (i.e., the salt substitute) and promoting its use within the intervention villages. The SALT project also had two different types of field staff: one responsible for the monthly distribution of the Salt Liz in the villages and the other for collecting health outcomes measurements (e.g., blood pressure) every 5 months. Additional intervention components are further described in Table 1.

**Table 1:** Description of the intervention components.

**Component**	**Description**
Salt substitute (branded as Salt Liz)	The study provided Salt Liz to the participants in their respective homes. Salt Liz did not need to be soaked in water to use it, it no longer had impurities. It saved them a step or effort before cooking. In addition, the jar allowed them to keep the salt close to their kitchen. The replacement of salt in each new village (step) was planned to happen every 5 months. The intervention considered delivering the salt to families, as well as to owners of small shops, bakeries and community kitchens, and food vendors including street vendors and restaurants. This approach was used to guarantee a full replacement of salt in the entire village. The delivery was done every month, and the amount varied according to each family. Additional Salt Liz packets were also made freely available during the study period in case any household required additional salt
“*Amigas de Liz*”	Members from the communities that work together with the research team. The objective of the “*Amigas de Liz*” was to be local product promoters of Salt Liz. Also, they developed and organized joint work with the research team to monitor the consumption of Salt Liz and conducted entertainment educational activities. They invited villagers to the entertainment educational activities. These women were asked for 2–4 h per week to dedicate to the project and they received training to do these activities. They were the sale force team of the Salt Liz
The Salt Liz spoon	The salt substitute was provided along with a spoon that introduced the right amount of salt that should be used to cook for each member of the family (half a spoon per person; Supplementary material 1)
Entertainment educational activities	These activities included bingo, raffles, quiz games, and the Healthy Dish competition. The objective of these activities was to promote the use of the Salt Liz, the use of the right amount of salt, and the knowledge about the benefits of the Salt Liz

The complex intervention was piloted in one village in Tumbes before full implementation. As part of the pilot, the messaging and tools used in the educational entertainment activities, a component of the social marketing campaign, were also tested.

### 2.3. Process evaluation study design and participants

The process evaluation of the SALT project primarily used a qualitative approach focusing on data gathered from semi-structured interviews of women in four out of six participating villages. A total of 15 intervention participants per village were recruited at random from a list and invited to participate in a one-on-one interview. Participants were recruited from two population groups: (a) women with a diagnosis of hypertension and (b) women without hypertension divided into the age groups of 18–34 years and ≥35 years to evaluate if age was related to decision-making capacity around food preparation. The interviews were conducted in March 2016. Additionally, data about Salt Liz consumption per household was collected at the beginning of the project only in one of the intervention villages and was used to estimate the amount consumed per individual (mean values).

Given the stepped wedge design of the trial, intervention villages were introduced to the salt substitute at different points in time during the study timeframe. By the time the interviews took place, the Salt Liz had been in a village for 23 months (village 1) while in another for only 2 months (village 4).

### 2.4. Procedures

A semi-structured interview guide was designed by an anthropologist (MAP) and a psychologist (FDC), both with expertise in qualitative research. The guide was not designed as a process evaluation tool. Instead, interview questions focused on understanding the experiences of Salt Liz consumers including how participants and members of their household adopted the salt substitute, how it was used in cooking, and reasons for continued use or discontinuation of Salt Liz. Interview questions further explored individual and household perceptions and opinions related to the Salt Liz and regarding several intervention components, such as the “*Amigas de Liz*,” the Salt Liz spoon, and the entertainment educational activities described in Table 1. Interviews were conducted by two anthropologists inside the participants' homes after receiving verbal consent.

Data on household salt consumption was collected in one village using a standardized scale; salt packages were weighed for three consecutive days at the beginning of the project.

### 2.5. Data analysis

Participant interviews were transcribed verbatim and MLP reviewed two transcripts while listening to the original audio to check for transcription accuracy. MLP and AD developed the codebook using a deductive approach (18). They read a subsample of five transcripts and identified the themes discussed by participants and assigned a code to each of them. This initial codebook was further complemented with codes that allowed the capture of factors proposed by the Medical Research Council (MRC) Framework for process evaluation (context, mechanism of action, and implementation outcomes) to understand how the intervention was implemented (12).

Five additional interviews were double-coded (MLP and AD). The list of codes was updated, and the researchers ensured that their criteria for coding interview segments were aligned. Later, each researcher coded the remaining interviews independently, using Atlas.ti version 8.0 (19). To ensure quality and consistency in the coding process, MLP and AD met every seven interviews to update the list of codes and/or address any doubts or challenges encountered. Once all interviews were coded, results were organized using the following MRC framework variables: context, mechanism of action, and implementation outcomes, such as acceptability, fidelity and adoption, perceptions, and feedback. Table 2 shows the definitions of the MRC framework variables observed.

**Table 2:** Medical Research Council Framework for process evaluation.

	**Definition**
Context	Identification of contextual factors that impacted the implementation of the SALT Project
Mechanism of action	Exploration of how the Salt Liz salt substitute became incorporated into the daily lives of community members and how it achieved expected changes
**Implementation outcomes**	
Acceptability	Satisfaction with the salt substitute and which factors play a role to improve the acceptability of the product
Fidelity and adoption	To what degree an intervention was implemented as it was prescribed in the original protocol or as it was intended by the program developers? In contrast, adoption is the degree to which the intervened population incorporates the intervention. Measures of the fidelity and adoption of the exclusive use of the salt substitute by community members, the use of the Salt Liz spoon, the “right” measurement of salt, and the amount of the salt substitute distributed were considered
Perceptions	Intervened groups' perceptions toward the intervention components, such as the “*Amigas de Liz*” and the education entertainment activities
Feedback	Intervened groups provide feedback to improve the intervention implementation

To estimate the consumption of the Salt Liz per individual (mean values), the average of the three consecutive days for each household (203 households) was calculated. These estimated values were added together and then divided by the number of members in the villages (790 people).

### 2.6. Ethics

The project was approved by the Institutional Ethics Committee of the Universidad Peruana Cayetano Heredia and Johns Hopkins University, and each participant provided written consent to use their data in ancillary studies including this process evaluation. Additionally, participants provided verbal consent to be part of the process evaluation study.

## 3. Results

### 3.1. Participants' sociodemographic characteristics

In total, 60 women with and without hypertension were included with a median age (p25–p75) of 66 (53–70) and 35 (29–50) years, respectively. Of the 60 participants, 14 participants reported having a family member with hypertension.

### 3.2. Context

Four primary contextual factors that played a role in implementation outcomes were identified: social characteristics and interpersonal interactions, food preparation and the use of other types of seasoning, the quality of the previously used salt, and the prevalence and knowledge of hypertension. Further information related to these contextual findings is outlined in Table 3, and Supplementary material 4 contains several quotes related to context.

**Table 3:** Context findings.

**Characteristics**	**Findings**
Community characteristics—who cooks and community interactions	The formative phase of the SALT project identified adult women (regardless of age) as the ones in charge of food preparation and as the decision makers of what is consumed by whom in their household. Subsequently, they became the target audience of the SALT Project's social marketing campaign. Additional analysis of the formative phase found that intervention villages are close-knit communities and their residents have known each other and lived there for years. Community closeness was exemplified in interviews when participants described how they would discuss the Salt Liz with neighbors and would share their opinions and experiences as well as hear the opinions and experiences of their neighbors and others in the neighborhood. Community characteristics of the villages played an important role in shaping aspects of the social marketing campaign, such as the recruitment of local women within each intervention villager to act as “*Amigas de Liz*” and help promote active participation from fellow residents in educational entertainment activities
Food preservation and use of other seasonings	Participants described certain food practices taken due to the lack of refrigeration. In total, 27 participants described using salt to preserve meats and fish. A total of 36 participants made or purchased “aliño,” a type of food seasoning in which salt is often added to it for flavoring and preservation. Furthermore, 28 participants mentioned adding salted food seasonings (Sibarita and Ajinomoto, see Supplementary material 3) in combination with table salt. Understanding the role of salt in the intervention villages allowed us to see what other additional sources of salt are consumed by participants, and to what extent it is possible to replace regular salt with the salt substitute and reduce the amount of dietary sodium intake in a household
Quality of regular salt previously purchased	There was consensus that the physical characteristics of the regular salt previously purchased were inferior to those of the Salt Liz. Most participants described the characteristics of regular salt as coarse and as leaving behind residual material akin to sand or dirt in prepared meals
Prevalence of hypertension and knowledge of causes	Of the 60 participants interviewed, 30 reported having hypertension and/or having a member of the household with a diagnosis of hypertension. The focus groups conducted in the formative phase of the SALT trial found that hypertension is known by villagers and recognized as a medical condition that negatively impacts an individual's health. However, stress and anxiety were identified as the main causes of hypertension. The awareness of hypertension in these local communities but the lack of knowledge of how dietary sodium consumption plays a role in hypertension informed the development of the educational campaigns in the intervention

### 3.3. Mechanisms of action

The process evaluation identified three mechanisms of action factors that helped incorporate the Salt Liz into daily use at the beginning and continue its use throughout: removing regular salt at the start of the intervention, adjusting to the change in taste and flavor, and understanding and experiencing health benefits. Supplementary material 4 shows several quotes related to mechanisms of action.

#### 3.3.1. Removal of regular salt from households and replacement with the Salt Liz

At the start of the intervention, field staff visited participating households and removed their regular salt and exchanged it, free of charge, with the Salt Liz. This removal and immediate replacement had the purpose of promoting the exclusive adoption and use of the Salt Liz in daily life. After this initial handoff, field staff continuously supplied households with the free salt substitute through monthly home drop-offs. As a consequence, most participants mentioned no longer had regular salt stored in their kitchens since its removal. In addition, several participants shared the perspective that people in their village solely used Salt Liz. Several participants described that regular salt was no longer available at their local grocery store. One store owner, a participant in the intervention, stated she got rid of bags of regular salt in stock because her customers no longer purchased this salt. Even when participants saw regular salt sold in stores, they found little reason to purchase or consume the product.

#### 3.3.2. Taste adjustment to the taste of the salt substitute and meal preparation

Initial impressions about Salt Liz revolved around its taste and the flavor it provided to prepared foods. Most commented that Salt Liz did not have the same “salting capability” as regular salt and that they noticed a change in the flavor in their foods. Several participants described how they and/or other household members initially went through an adjustment period. Part of adjusting to the salt substitute included getting accustomed to eating foods with lower sodium content and learning, through trial and error, the amount of Sal Liz it takes to give meals their desired flavor. In other words, participants had to recalculate the amount of salt needed in preparing day-to-day foods since the Salt Liz was a new type of salt considered to have different flavoring capabilities. Overall, participants and household members in all four villages that reported initially tasting a difference between regular salt and Salt Liz took about 2 weeks to adapt to the salt substitute's flavor and salting abilities. Those who expressed having been accustomed to eating foods containing little salt or flavored seasonings did not express noticing taste differences in their meals when they changed salts.

#### 3.3.3. Understanding and experiencing health benefits from the salt substitute

Participants learned from the social marketing campaign, project staff, and word of mouth (neighbors) that Salt Liz is a healthy alternative to regular salt. Several participants described the Salt Liz as being “good,” “healthy,” and that “it does not do any harm.” When asked about the positive health effects of the Salt Liz use, participants explained that it can help with diabetes and cholesterol problems. Most participants correctly understood that Salt Liz can help control blood pressure, an understanding shared by participants with or without hypertension. Understanding of health benefits from consuming Salt Liz was not focused solely on knowledge but also on the experience of participants while using the salt substitute. Some participants shared that general physical aches and discomforts, such as headaches, knee aches, and tiredness, disappeared and have not returned after switching from regular salt to Salt Liz. Some also attributed unrelated health outcomes, such as weight loss and relief of digestive issues, to the Salt Liz. Importantly, participants with hypertension shared how the use of the Salt Liz has alleviated physical symptoms. One participant shared that she no longer feels agitation or heart palpitations and no longer needs to see their doctor on a weekly basis; several others have also shared that they no longer experience headaches, dizziness, and tiredness. Several participants without hypertension expressed that family members with hypertension have regulated blood pressure since switching to Salt Liz. The experienced and perceived health benefits of the Salt Liz served as a motivating factor for participants to continue their use of the salt substitute as they were adjusting to its taste and flavoring capabilities, including when there was pushback from members of their household (e.g., spouses, children) who may have hypertension or other health problems.

Several participants attempted to explain the reasons for the positive effects of the Salt Liz on health, leading to statements about potassium, sodium, and iodine intake. The accuracy and understanding of these elements widely varied across participants. While most participants understood that the salt substitute helps control blood pressure, only a few understood the specific roles of sodium and potassium in hypertension management. Supplementary material 5 outlines participants' understanding of sodium, potassium, and iodine and their relationship between health and hypertension. Supplementary material 6 provides a description of common explanations given by participants about why Salt Liz is healthy.

### 3.4. Implementation outcomes

#### 3.4.1. Acceptability of the salt substitute

Acceptability of the Salt Liz in daily life was high among participants. Learning about Salt Liz's effects on health influenced acceptability among participants. Participants and other villagers learned and received confirmation of the salt substitute's health effects through the different social marketing campaign components, project field staff, conversations about Salt Liz among each other, and, for some participants, from medical providers. In this latter case, a few participants with hypertension asked their doctors about Salt Liz's ability to help them manage their blood pressure and their doctors supported their use of the salt substitute. Conversely, given that 25% of Salt Liz's composition consists of potassium, participants also learned from these same sources that those with kidney problems would not be able to consume the salt substitute. As a result, participants also mentioned knowing some villagers who did not participate in the intervention for this reason.

Participant interviews revealed that project field staff played a role in promoting the acceptability of the salt substitute. The tasks of field staff in the intervention were to visit households and take health measurements or drop off Salt Liz. However, these household visits unintentionally led to opportunities for the reiteration of the salt substitute health benefits and encouragement of continued use of the Salt Liz. Participants described asking questions and engaging in conversation with field staff about the Salt Liz during these visits.

Beyond health, participants in the four villages accepted the Salt Liz because it was a higher quality salt. Compared to the regular salt previously used, most participants noted that Salt Liz had a finer texture, did not contain impurities, and dissolved easily without leaving behind residue in prepared meals.

Several participants mentioned accepting Salt Liz and continuing its use because not having to pay for salt helped cut down household costs. Furthermore, participants felt motivated to participate in an opportunity their village was chosen for and was not available everywhere. Positive experiences with prior research projects that occurred in their villages created trust among several participants for becoming involved in the SALT project and accepting the salt substitute. Participants from two villages mentioned a previous positive experience with a project of our university related to cysticercosis (20). Supplementary material 4 contains quotes related to acceptability as well as quotes related to other implementation outcomes.

#### 3.4.2. Fidelity and adoption

These outcomes are presented together because there is a certain overlap between them. The definition is available in Table 2.

##### 3.4.2.1. Exclusive use of the salt-substitute

A total of 56 participants out of the 60 participants interviewed used the Salt Liz exclusively for all household and commercial food preparation (e.g., preparing cheeses to sell at the market, etc.). Of these 56 participants, not all immediately replaced regular salt with Salt Liz. A few participants initially combined the two salts together in food preparation and stopped after becoming accustomed to the taste of the Salt Liz or when they ran out of regular salt. Exclusive use of the Salt Liz by participants also depended on their households and businesses receiving sufficient quantities of the salt substitute to last the entire month. Almost all participants stated that they received enough salt for the entire month, with some adding that at times they had Salt Liz left over. One participant shared running out of the Salt Liz before the end of the month and having to use regular salt until the next month's delivery. In the instances where a participant's household continued their use of regular salt after receiving Salt Liz at the beginning of the intervention did so because their household could not get accustomed to the taste or because of health reasons. One participant whose husband had kidney disease prepared meals for him using regular salt.

##### 3.4.2.2. The use of the Salt Liz spoon

The Salt Liz spoon was provided as a way to measure the right amount of salt to use in food preparation based on the number of family members in a household (half a spoon per person). Half of the respondents mentioned using the spoon regularly (30/60), a small group said they sometimes used the spoon (5/60) and just under half of the respondents did not use the Salt Liz spoon at all (25/60). Those who did not use the Salt Liz spoon used larger spoons instead (such as a stirring spoon) (14). Several mentioned they are accustomed to adding salt to taste or based on experience (3) and the rest did not specify. Participants who used the Salt Liz spoon explained that it helped them gauge the right amount of salt that should be consumed in a meal. They also praised the aesthetics of the spoon, noting that it was “comfortable and beautiful.” Infrequent users mentioned primarily using the Salt Liz spoon when cooking for a small number of people, noting that it was too small to use when preparing large quantities of food (e.g.; to sell) and when scooping and stirring Salt Liz from its container to a pot. Consequently, some participants mentioned that even if they did not use the Salt Liz spoon, they tried to estimate the equivalency of their large spoons to the number of Salt Liz spoons.

##### 3.4.2.3. The “right” measurement of salt

Participants self-reported the amount of salt they added to foods, and these self-reported values were described either in the Salt Liz spoon measurements or in other formats such as utensils and kitchen spoons or pinches of salt. Therefore, the amount of salt consumed by participants in their meals during the intervention was estimated by developing a reference guide of the different types of spoons (Salt Liz spoons, soup spoons, or plastic spoons. See Supplementary material 2), as well as by the number of people in a household (e.g., one Salt Liz spoon for two people) and/or a given quantity of food (e.g., one kilo of rice). Amounts reported were categorized as either consuming more than the recommended amount or consuming equal to or less than recommended.

Approximately half of the people interviewed (30/60) consumed more salt than recommended by the project (half a spoon per person), and one-third reported using the recommended amount or less salt (19/60). A smaller group did not report an exact amount but mentioned that they were “eyeballing.” When comparing the number of participants that used the Salt Liz spoon with the amount of the salt substitute consumed, it shows that the use of the Salt Liz spoon does not seem to make a difference. Even when the Salt Liz spoon was used or when participants tried to estimate the correct intake related to other measurement tools (i.e., other spoons), it was still likely that users did not follow the recommended salt intake.

##### 3.4.2.4. Amount of the salt substitute distributed and consumption per individual

The most common amount of the Salt Liz field staff distributed to households was 2 kg per month for personal consumption and the median number of family members in a household was 4 (3–5). The smallest monthly amount received by households was 1 kg while the highest was 5 kg. In addition, eight women reported receiving more Salt Liz (in most cases, 2 kg) for their food preparation business. Three families received an additional 1 or 2 kg of the Salt Liz to use for feeding livestock.

The average value (SD) of salt consumption per household in one intervention village was 55.3 gr ± 32.7, the median (p25–p75) was 48.1 gr (33.0–67.0); when added together, the salt consumption was 11,270 gr, and consumption per individual (mean value) was 14.3 gr.

#### 3.4.3. Perceptions surrounding the social marketing campaign components

##### 3.4.3.1. “Amigas de Liz”

The “*Amigas de Liz*” (Liz's friends) were community members recruited to promote the consumption of the Salt Liz in their village and motivate villagers to participate in events and activities related to the SALT project. Participants were asked if they have heard of and could identify the “*Amigas de Liz*” in their village, and, in most cases, participants identified them by their first name. Some participants considered the “*Amigas de Liz*” as being part of the “club” or knew them as “the people with the blue t-shirt” or as health promoters. Participants also recognized “*Amigas de Liz*” as their neighbors. “*Amigas de Liz*” was also viewed as community members who promoted the “Liz” brand and supported project field staff. One participant mentioned that she was invited to become one of the “*Amigas de Liz*” in her village but turned down the offer because it would have required her to exert physical effort such as carrying materials around.

Participants were able to enumerate the responsibilities and actions undertaken by “*Amigas de Liz*” and activities they assisted in or led. These activities included the following: recruiting neighbors to participate in the Healthy Dish competition, conducting door-to-door visits and inviting households to various entertainment educational activities (i.e., raffles, bingos) that took place in the village public square, and distributing materials (e.g., calendars, flyers, and leaflets) depicting the “Liz” brand and educational information. The most identified educational material by participants were leaflets shaped as a traffic light that indicated which foods were high in sodium (red) and low in sodium (green). Finally, participants mentioned that “*Amigas de Liz*” taught villagers how to consume a balanced diet, what is the correct amount of salt to use, and which foods are rich or poor in potassium. One participant knew that “*Amigas de Liz”* were trained to orient villagers about how to use Salt Liz and what are its benefits.

##### 3.4.3.2. Entertainment educational activities

Some participants mentioned that they only went to see the Entertainment Educational Activities (EEA), but did not participate, others mentioned that they participated actively, and the rest mentioned that they did not go. Those who did not go cited work, household demands, schedule conflicts (e.g., Sunday service), and not being aware that events in the plaza were related to the Salt Liz as the main reasons for not attending EEA. Participants who participated as spectators shared that they felt too embarrassed to partake in activities, that they worried they would not be able to answer the questions, or that they had brought their children along and thus preferred to “just watch.” Regarding the “Healthy Dish” competition, some did not participate because they did not cook well or because they could not afford to buy the ingredients (fish is expensive).

In general, participants enjoyed attending and participating in EEA because they found them fun, won prizes, and had a chance to relax and laugh. However, some participants shared complaints regarding the way prizes were awarded in the “Healthy Dish” competition. Some did not agree with whom the judges determined to be the winners and others thought that the prize (a basket filled with household food staples) should have been shared among all participating contestants instead of it being awarded to one person. However, participants did not share complaints over other events hosted by the “*Amigas de Liz*,” such as bingo, raffles, or educational quiz games.

#### 3.4.4. Feedback

Most participants said they would like to buy the salt substitute if it becomes available for purchase (56/60). On average, participants are willing to pay 2 PEN (0.54 USD) per kilo (double the price of regular salt); the minimum monetary value reported was 0.5 PEN (0.13 USD) and the maximum was 6 PEN (1.61 USD).

Participants shared how ingrained Salt Liz was in their community by stating “everyone is using it” and “now they can't do without.” Some participants would ask rhetorically or to the interviewer if (or when) there were plans to stop the program and discontinue the distribution of the Salt Liz.

Financial aspects and cost-savings came up in interviews with participants across different villages after the introduction and utilization of the salt substitute. Eight participants expressed concerns about no longer receiving free salt (referring to the Salt Liz) after the trial ended.

## 4. Discussion

### 4.1. Main findings

Using the MRC framework, several process evaluation components were identified that help explain the success of the SALT project, a salt substitute trial. Context-related elements that influence trial implementation included (1) social characteristics and interpersonal interactions in the intervention villages, (2) the place where villagers eat and the person responsible for preparing food, the poor quality of the regular salt consumed by villagers prior to the intervention, and the prevalence and knowledge of hypertension.

Just as important as these contextual elements were the mechanisms of action that facilitated the uptake and day-to-day use of the Salt Liz: (1) Retrieving and replacing the regular salt with the Salt Liz in participating households, (2) adjusting to the taste of the salt substitute and foods prepared with it, and (3) understanding and experiencing health benefits while using Salt Liz.

The social marketing campaign succeeded in promoting the use of the salt substitute in intervention villages and in spreading the message of the Salt Liz as a healthy alternative to regular salt and that it is helpful in preventing or controlling hypertension. However, most participants could not explain the differences in composition between Salt Liz and regular salt and how it relates to blood pressure.

Overall, our study shows that Salt Liz was accepted by the villagers where the trial was implemented. Factors that facilitated such high acceptability included the following: (a) the perceived high quality of the Salt Liz in comparison to regular salt previously consumed, (b) the fact that Salt Liz was free and distributed directly to participants' homes according to need, and (c) the trust in the institution who implemented the trial (“*Centro de Salud Global,” Universidad Peruana Cayetano Heredia*) as a result of a previous positive experience with a project related to cysticercosis led by the same university.

Although we do not have exact measurements of the Salt Liz consumption, we have three indicators that show that the suggested measure of the Salt Liz was not used. One indicator was the consumption of salt per individual (mean value) of 14.3 gr found in one village, a value nearly three times greater than the World Health Organization's recommendation of 5 gr per day (21). The second indicator was the self-reported poor use of the Salt Liz spoon, while the third indicator was the results of the urine analysis showing an increased potassium level and unchanged sodium level. A potential explanation for this third indicator is that subjects consumed more Salt Liz to obtain appropriate food taste. These indicators provide evidence that potassium played a preponderant role in lowering blood pressure.

### 4.2. Comparison with other studies

One process evaluation (22) of a clustered RCT (7, 23) was identified; this cRCT in rural China implemented a salt substitute free of charge comprised of the same components as Salt Liz and targeted households where someone had suffered a stroke or was at higher risk. The implementation of this study was through primary care physicians. The process evaluation found high adherence to the salt substitute and identified some similar factors with our study such as predominance of home cooking, high acceptability of the salt substitute taste, and poor knowledge about how the salt substitute works. An additional aspect that was identified and discussed in the Chinese study was that their salt substitute was provided free of charge. Previous evidence found that studies providing salt substitutes at no cost had larger effect sizes compared to studies where participants had to buy the salt substitute (24–27). This is a key element to take into consideration if the scaling up is going to focus on low-resource settings where having a salt substitute that is more expensive than regular salt might deter its uptake.

Another global study identified barriers and facilitators to implementing reduced-sodium salt interventions at the population level from the perspectives of different stakeholders (28). Barriers identified were poor availability of the product in most countries, limited evidence on health outcomes among patients with kidney disease, and the possible use of greater amounts of reduced-sodium salt affecting its effectiveness. Other barriers described were higher prices in comparison to regular salt and higher costs of production, the difference in taste, and low demand for reduced-sodium salts. Implementation facilitators were evidence of efficacy/effectiveness from trials, reduced retail prices, tailored health education, and mass media campaigns. The current study faced some of the described barriers through the implementation of the SALT project. The problems with the taste were overcome after two weeks, and participants expressed willingness to keep using the salt substitute even if the price was twice the one of regular salt. The evidence from our study and other studies identified (29) in the literature review showed that the positive health outcomes can be sustained and scaled up if different community stakeholders and decision makers get involved to address barriers and integrate the salt substitute as a public health intervention.

### 4.3. Relevance for future implementation or scale-up

There are five main aspects relevant for future implementations of salt substitutes: improving tools that guide reductions in salt intake, keeping simple messaging about the benefits of using the salt substitute for blood pressure control, addressing other sources of additional sodium intake, optimizing the promotion of salt substitute intake during the initial period, and the importance of providing salt substitute free of charge.

Utilizing the correct measurement of salt in food preparation is an important part of maintaining a low-sodium diet. However, less than half of the participants reported consistent use of the Salt Liz spoon, indicating poor utilization. Future interventions will need to consider how to design salt measurement tools that are versatile enough for everyday cooking and kitchen use (i.e., scooping and stirring). The design of the Salt Liz spoon was based on formative research data (16), but given explanations of its poor utilization during the trial phase, further research or a pilot of the tool could have been conducted to test its usability.

The overall message that the salt substitute helps control hypertension was understood by most participants. However, participants did not understand how the salt substitute helps manage or prevent hypertension or the impact of sodium and potassium on blood pressure and the role of iodine in salt. The aim of the social marketing campaign was to introduce the salt substitute independently of the participants' understanding of its mechanism of action on blood pressure control. Thus, if future interventions aim at participants to clearly understand the benefits of potassium in hypertension control without confusing it with sodium or iodine, they need to identify culturally appropriate pedagogical approaches.

Efforts to reduce dietary salt intake need to consider how participants may inadvertently consume additional salt found in other food and flavoring sources. The contextual analysis in this process evaluation found that the use of seasonings such as “*sibarita*,” “*aliño*,” and “*ajinomoto*” (monosodium glutamate) and food preservation techniques can be a source of additional salt consumption. Similarly, a study in China (15) reported the use of pickled foods, and another study in China and Indonesia also reported the use of monosodium glutamate (29).

Implementing social marketing strategies can be effective in sustaining the consumption of a salt substitute product during the initial period as a consumer becomes accustomed to its taste and how it enhances flavors. Social marketing strategies of introducing and promoting a new product in a community become more important for interventions where it is not possible to completely replace the old product with the new one, as it was possible in the SALT project, or when the old product is equal in quality as the new product.

From our findings, people were motivated to try and continue with the salt substitute because it was provided to them at no cost. Individual finances and the economic status of a community can factor into the uptake of a newly introduced product. Several participants stated that the advantage of Salt Liz is that it was free. While the regular salt itself is not an expensive product (e.g., 0.13 USD) in the region of Tumbes, it is widely and frequently used. Therefore, it becomes advantageous for households with less financial means to exchange a product they pay for with one that is free and serves the same function. It becomes uncertain how a community would respond to a salt substitute if they have to pay for it. Currently, commercial low-sodium salts have a composition between 0 and 88% of NaCl and are priced between 1.1 and 14.6 times higher than regular salt (30).

Another consideration in the replacement of regular salt with a salt substitute is the availability of potassium. In the context of Peru, potassium would need to be imported from other countries. If the deployment of salt substitutes wants to be scaled up in the future, the costs of production and also potential costs to consumers would need to be strongly considered.

### 4.4. Strengths and limitations

This study contributes to the literature on process evaluation for complex interventions involving salt reduction strategies. It shows that some elements that enabled the uptake of the salt substitute (such as the trust in the university implementing the trial and complete removal of the old product) might not be possible in other contexts. There are few publications related to salt substitution interventions. Utilizing the MRC framework allowed us to retroactively identify and explore process evaluation components of this complex salt substitution intervention, leading to a better understanding of which and how different study components factor into successful project outcomes.

The evaluation of the social marketing strategies used by the SALT Project is a strength of this study. Social marketing was used to promote the uptake of a salt substitute through branding, educational entertainment, and community outreach. The evaluation of a social marketing campaign allows the exploration of how different campaign components worked in the field and are perceived by those on the receiving end. Ultimately, this can help increase knowledge on how social marketing can be used to promote the uptake and continued use of products that improve health outcomes.

A limitation of this study is that not all process evaluation components of the MRC could be applied to this study due to the lack of appropriate data. One example is that the intervention did not take an initial baseline measurement of the amount of regular salt consumed by participating households and we could not retrospectively measure changes in salt consumption during the intervention and at different points in the intervention (e.g., the initial period). However, a random subgroup of participants (*n* = 600) provided urine samples for analysis both at the start and the end of the intervention. We found no difference in the levels of sodium measured at both moments; however, higher levels of potassium at the end of the trial were found compared to the baseline, which ensures the salt substitute consumption.

Additionally, measurements related to the Salt Liz consumption per individual, as well as the quantity of the salt substitute used in meals and consumed food portions relied on approximate and self-reported data, respectively. In the case of the Salt Liz consumption, measurements taken during the first 3 days of the project in one village were available, and the value of 14.3 gr can include some bias; one is related to the number of participants because we do not have the exact number of members per household. Second, even when it was specified by fieldwork staff that some packages were for cooking and other packages for other uses (e.g., preparing food items to sell), it is possible that some households ended up using the same package for different purposes. However, the urine analysis from the subgroup mentioned found that the average salt consumption was 11 gr (9), which is not too far from our finding of 14.3 gr.

In the case of the self-reported use of the salt substitute, participants often would state using the Salt Liz spoon or a big spoon to measure the quantity of the salt substitute when cooking different meals and the number of scoops used per prepared dish. However, in some situations, quantifying precise amounts proved difficult as some participants would report consuming “one fistful” of rice or “eye-balling” the amount of salt substitute added to certain dishes. The imprecise values of how much salt is added while cooking is a limitation of these types of studies because people normally tend to add salt and other condiments until acquiring the desired taste (“al gusto” in Spanish), not necessarily focusing on how much salt was added. Also, another type of reporting bias is the potential social desirability bias because, during the interviews, participants associated the interviewers with the project.

We did not include the challenge of measuring with high precision the actual daily consumption of the Salt Liz in the study population during the 24 months of the project as part of the study objectives. Although such measurement is important and should be a task for future studies, it would have involved the logistical challenge of having fieldwork staff visiting households daily for a certain period using appropriate calibrated scales to weigh the salt containers and record the difference in weight found; additionally, we would need to know how many people consumed the prepared food in each household and whether the salt was used for other purposes. Methodologically, there would still be a question as to whether the consumption during the evaluation period is representative of real consumption. We also do not know whether a close measurement would have been an intervention in itself and would have changed the true consumption pattern of the households as they perceived a closer monitoring of salt consumption. For future studies, it is important to think about how to overcome these challenges.

## 5. Conclusion

This process evaluation identified aspects of intervention components that enabled a successful trial, such as a salt substitute free of charge, the idea that Salt Liz was healthier in comparison to regular salt, the perceived “high quality” of the Salt Liz in comparison to regular salt, and the previous positive experience with a project from the same university. Furthermore, the social marketing campaign succeeded in incorporating the salt substitute into the day-to-day cooking by participants in the intervention villages. The poor use of the Salt Liz spoon is a lesson of this intervention that can be improved in future studies.

## Data availability statement

The raw data supporting the conclusions of this article will be made available by the authors, without undue reservation.

## Ethics statement

The project was approved by the Institutional Ethics Committee of the Universidad Peruana Cayetano Heredia and Johns Hopkins University, and each participant provided written consent to use their data in ancillary studies including this process evaluation. Additionally, participants provided verbal consent to be part of the process evaluation study.

## Author contributions

Study design of the qualitative study and data collection: MAP and FDC. Study design of the main study: VPL, ABO, MC, JM, and FDC. Data analysis: MLP, AD, and SPL. Data interpretation: DB, MAP, VPL, ABO, MC, FC, PP, JM, and FDC. Manuscript writing: MLP and AD. Manuscript approval: All authors. All authors contributed to the article and approved the submitted version.
