# Supplementary material for: Implementation of a salt substitute intervention using social marketing in resourced-limited communities in Peru: a process evaluation study
Source: Front Public Health. 2023 May 19;11:1068624. doi: 10.3389/fpubh.2023.1068624 (PMC10235695; doi:10.3389/fpubh.2023.1068624)
Supplement: Supplementary file 1 [file Table_1.docx]

# Supplementary Material

## Supplementary Material 1. Picture of the Salt Liz product and the spoon


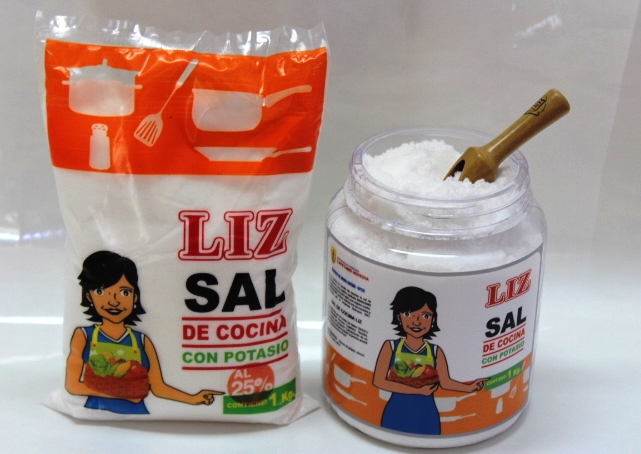


## Supplementary Material 2. Equivalency table of salt measurement

| Equivalency Table | |
| --- | --- |
| Self-reported Measurement | Equivalency |
| 1 stirring spoon: | Equivalent to 3 teaspoons of Salt Liz. |
| 1 plastic teaspoon: | Equivalent to 1 teaspoon of Salt Liz. |
| 1 kilo of rice: | Equivalent to 5 servings of rice, or one serving per person for 5 individuals. |
| **Recommended serving by the study for one person: ½ teaspoon of Salt Liz per person.* | |

## Supplementary Material 3. Seasonings used by study population

**Aliño**

Generally defined as, “salsa, condiments or other ingredients added to foods in order to enhance taste and flavor.” However, in Tumbes, aliño indicates a preparation of achiote, sibarita, garlic, oil and salt. There can be variations and the quantities of ingredients are defined by those that prepare it.

**Ajinomoto**

“Sodium Glutamine, in white powder, that is used as a condiment for enhancing flavor of certain foods, especially meats”

**Sibarita**

Commercial brand name of a seasoning that includes a mix of panca, achiote, salt and condiments.

## Supplementary Material 4 Quotes according to the components of the Medical Research Council Framework for Process Evaluation

| **Components MRC Framework** | **Quotes** |
| --- | --- |
| **Context** | **Note**: Interviewer (I), Participant (P)  Information inside brackets provide additional context to quotes cited.  I: Who decides what to cook?  P: What I prepare, he… he eats (laughs).  *Female, 66 years old, has hypertension*  I: And do you salt all meats with Salt Liz also?  P: Yes, meat, fish, with that salt. We do not consume any other type of salt.  *Female, 50 years old, no hypertension*  I: ¿Do you use aliño?  P: Yes, also aliño  I: Do you prepare it yourself or do you buy it?  P: No, I prepare it, my aliño. I mash it.  I: Do you add Ajinomoto?  P: Yes.  I: Sibarita?  P:  I tell you I don’t, if I already use aliño, I do not use any Sibarita.  …  I: And have you tried your meals without aliño, without Sibarita?  P: No.  *Female, 66 years old, has hypertension*  [comparing Salt Liz to regular salt]  P: … So, the one brought to me came out to be pretty good because the other salt was [unintelligible] and it would have sand, it was dirty.  I: In other words,  it had more… more impurities.  I: Yes, more impurities, yes. Even if it looked pretty clean.  *Female, 65 years old, husband has hypertension*  I: What advantages do you find in Salt Liz that make you state that?  I: Like I said, in controlling my, well, I control my blood pressure. Because, since they told me I have high blood pressure, they… they prescribed me medications and so… I have to take them for the rest of my life.  *Female, 66 years old, has hypertension*  I: And the doctor that you see, did you tell him that you use Salt Liz?  P: Yes, it just so happens that my daughters are chatterboxes, so since the beginning. We had to go to the doctor and they brought along the packet so he could see it. ‘This salt is fine’ - he said -  It is good…. Continue to use it’ - he said - ‘this salt is good’ And that was all he said.  I: Where is the doctor from? Tumbes?  P: Yes, from Tumbes, From around there.  *Female, 68 years old, has hypertension* |
| **Mechanism of Action** | I: And when you started cooking with Salt Liz, for example, the first time you made your meal with Salt Liz, did you tell your family that you were using Salt Liz or did you not say anything?  P: Well, yes, because it was a project that had a lot of  advertising around it and everything… and at the very least it was known that they were going to bring us that salt, and that we were no longer going to buy the other salt. But rather, we were going to only use Salt Liz.  I: And what did your family say?  P:  Nothing, some were fine, they said let’s try it… Since there was so much promotion, at least, well, they wanted to opt for using that salt… see what it was like…  I:  Did you think it was good marketing, what they did?  P: : Of course. Even now it is still happening. Sometimes there are cooking competitions of typical dishes; everything… And people come out… Well, yes they come out to… come out with their prepared dish to display… And only because of Salt Liz, nothing else…  I: Only Salt Liz does that.  P: Yes, only Salt Liz does that.  *Female, 34 years old, no hypertension*  I: And when you changed out the salt, did everyone [in your house] know about the change?  P: Yes.  I: How did you tell them?  P: So here there was a [community] meeting to bring over that salt. They [the household] already knew that it was coming. All the salt that was there before was taken, they left us the other salt. They already knew at home that they had changed out the salt.  I: Did you tell them something, to your husband and daughter, other than the meeting that took place, about the change of salt?  P: Yes, I told them that they were going to bring other salt because it was better. That there were a lot of people here with high blood pressure. And that this would be better for our health.  *Female, 43 years old, has hypertension*  I: Ah, so you’re saying that you did not think the taste was that great.  P: Yes, I didn’t think it was that good. But since we kept at it, and kept at it, and at it,  ahhh until we got, got used to it... and we are still with the salt.  *Female, 53 years old, has hypertension*  P: Just like… like with sugar… like… like Stevia, hm… almost similar. Sweetness, just like saltiness, yes.  There is a great difference between sugar and Stevia. And like that, almost similar… but now with the time that has transcurred in using the salt, I no longer find it… I am now used to that salt.  *Female, 37 years old, husband has hypertension*  I: And have you perceived any changes in your life since you started using Salt Liz?  P: At least for me, I think my blood pressure doesn’t rise as much as before. Before my pressure would rise a lot, now it doesn’t, almost never. So I feel that it has helped me a bit.  *Female, 28 years old, has hypertension*  I: And if you compare Salt Liz to the salt you used before, which do you find better?  P:I find Salt Liz is better.  I: And what are the advantages that you see in it?  P: For example, the regular salt has too much… it is all sodium, and Salt Liz instead, has potassium which helps us, you know, all of us. So, because of that I prefer to use Salt Liz.  *Female, 28 years old, has hypertension*  I: And what are the advantages that you find?  P: Well, in… in Salt Liz, the advantages that, is that…, it is healthy, yeah, healthy. Well, when I consumed the other salt, we [unintelligible]... I would get inflammation of my urinary tract…  and with this salt, I don’t know, I don’t… don’t. I no longer suffer from my inflammation.  I: And do you believe, let’s say, that you do not suffer from inflammation because of Salt Liz or because of another reason?  P: I: I don’t know, well, maybe… I don’t know why…. Before I would suffer a lot from… when I ate the other salt, it was that I ate… I would eat quite salty, I would eat super salty. But not anymore.  *Female, 32 years old, no hypertension*  I: And what motivated you to receive and use Salt Liz?  P: Because there was this… this with the salt and since the “Amigas” would say [unintelligible] it would not do harm, this other salt, and it would be better, yeah… well, and like how around here the ladies and young staff would come by… to promote the salt, so I signed up. Everyone here now uses that salt.  *Female, 74 years old, no hypertension* |
| **Implementation Outcomes** |  |
| Acceptability | I: And what advantages do you see in Salt Liz?  P: Well it is a bit whiter and finer, doesn’t contain much, doesn’t have what the other had, like the other salt that had sand. Yes, when we would make a salsa, something would stay behind… like sand it looked like... In this one no, this one is… normal.  *Female, 56 years old, has hypertension*  I: Is there… Do you find an advantage in using Salt Liz?  P: Well, more than anything is that it is free (laughs)  *Female, 38 years old, no hypertension*  I: And at any point have you thought about no longer using Salt Liz?  P: No, not at any point. Only if I die (laughs).  *Female, 56 years old, has hypertension*  I: Pleasant in what way?  P:  In… in its texture, you know? It has a finer texture, it is refined and… and salts normally, you know? Because there are, well, other salts where you have to add a lot. With this one, a little bit is enough. And also in the case of, for example, my parents say that this salt helps to control blood pressure, right? But no, I wouldn't know what to tell you, because we do not, hm… have high blood pressure. But yes, it is a pleasant salt. It doesn’t have a bad taste in foods. In other words, it doesn’t affect us in that sense, you know?  *Female, 39 years old, no hypertension*  P: This salt is practically made for hypertensive individuals, no? It seems to me that it was made with the purpose of saving lives because it is a salt that doesn’t, doesn’t harm anyone, no one… neither children, nor the elderly… does not harm in any way. It is a very good salt.  *Female, 70 years old, she and her husband have hypertension* |
| Fidelity | I: And for which foods do you use Salt Liz?  P: For everything the salt, in all my foods.  I: In all?  P: In all.. In soups, in legumes, in pretty much everything… the salt we have now… now with… We no longer use the other salt, only this one… Yes, yes.  *Female, 66 years old, has hypertension*  P: Yes, I have used the Salt Liz spoon… How do I explain it. At first I would use it, but when you are in a hurry, desperation gets a hold of you and time passes you by. And I have already, have compared the Salt Liz spoon to my spoon and I already know the measurements that I use. So when I have time, I use the Salt Liz spoon calmy. However, when I am in a hurry; since I already know the measurement to use, I just get my spoon. But provided that I try it, it is not that I just add for the sake of adding.  *Female, 34 years old, no hypertension* |
| Perceptions | P: … Mrs.[Name] comes over and talks to us. When she stops by, she… she tells us what they tell her in the chats, she comes over and tells us.  I: What things do you remember that she, more or less, has told you?  P: Well, they… they say that… we need to consume, consume the salt according to the measurement of the Salt Liz spoon, to consume a lot of… the traffic light… in the traffic light it says what is high in potassium, it tells us eat foods that are high in potassium… It tells us.  [Traffic light refers to a SALT project pamphlet passed out to villagers by the Amigas de Liz]  *Female, 32 years old, no hypertension*  I: And do you know what they do on behalf of Salt Liz?  P: Well, they… they only let us know when an event is held, that they will attend… in the events held. Just that, nothing else. Because beyond that, another thing no. They do not explain, do not give an explanation. Like I told you, I would not have been able to ask them what… where… I don’t even think they would know.  *Female, 66 years old, has hypertension*  I: And have they invited you to any activity so you can go to the plaza?  P: Yes.  I: And have you been able to go?  P: Sometimes, but usually not. I hardly have been able to because sometimes I work and can’t.  *Female, 31 years old, no hypertension*  I:You mentioned that not long ago there was an event.  P: Yes! The day before yesterday.  I: How did it go?  P: Regarding the dish presentation, yeah, the presentation of dishes flavored with the salt. Sometimes, the small raffles. So that is held by the salt… Salt Liz… is holding this… They are holding lots of events here.  I: And do people generally participate?  P: Yes, yes… everyone participates… For us it is a celebration when “Salt Liz” comes over. That is to say, you should participate, well, because they are giving us a lot of support because of the salt.  *Female, 74 years old, no hypertension*  I: Why do you like to go?  P: Because one can learn more, know more about the type of salt that we are consuming.  *Female, 34 years old, no hypertension*  P: Mainly because they always go. First the older women would attend, but now this last time… a lot of younger people have attended who wanted, well, to know how to make those semi-simple dishes. Because here when they showcase [dishes] they almost always say without frying, with nothing… and people go, a lot of them… The last time they made mackerel ceviche… And well, this is a town that always likes those things. When there is something good to learn, people show up.  *Female, 34 years old, no hypertension* |
| Feedback | I: And have you ever thought about no longer using Salt Liz?  P: Not even as a joke has it crossed my mind.  *Female, 68 years old, has hypertension*  P: No one will want to go through the inconvenience of getting sick over buying salt at a store for 50 cents. One that is dirty, has dirt in it. At times  it has even come with hair. Moreso for their children because babies are the ones that get sick more often.  *Female, 31 years old, no hypertension*  I: How much would you pay for Salt Liz?  P: However much it costs. Well because, how much would a packet be.  I: Let's imagine that it is twice as much as regular salt.  Regular salt is at one Peruvian Sol and Salt Liz would be at two Peruvian Soles. Would you pay one more Peruvian Sol for Salt Liz, or would you prefer, lets say, your wallet and pay only for the salt that is one Sol?  P: That's why I say, well, let's buy it. Well in anyways, we have to buy the same salt, the one to which we have already got accustomed to.  *Female, 66 years old, has hypertension* |

## Supplementary Material 5 and 6. Knowledge of Salt Liz and regular salt components and relationship to hypertension

### Supplementary Material 5. Participant knowledge of Sodium, Potassium, and Iodine

| **Sodium** | **Potassium** | **Iodine** |
| --- | --- | --- |
| Too much sodium causes hypertension | Potassium helps lower blood pressure  potassium can be harmful for individuals who suffer from kidney problems.  important to consume foods with potassium; necessary as part of a healthy diet | Important for salt to contain iodine  Iodine is necessary for thyroid function  Iodine is not good for health; the less consumed the better for your health  Salt Liz contains more iodine than common salt  Iodine is found in both common salt and salt Liz |

### Supplementary Material 6. Participant knowledge of Salt Liz

###

| Participant Response |
| --- |
| Salt Liz contains is a better salt because it contains less iodine |
| Salt Liz contains less chemicals |
| Salt Liz is made up of chemicals that are different from common salt |
| Salt Liz cannot be consumed by those with kidney illness |
| Salt Liz helps reduce hypertension, no further explanation as to how |
| Salt Liz helps reduce hypertension because it contains potassium |
| Salt Liz helps regulate other body vitals and functions (triglycerides, blood circulation, stomach aches) |
| Common salt does not contain potassium |
| Common salt contains more potassium than Salt Liz |
| Salt Liz contains less sodium than common salt |
| Both Salt Liz and common salt contain Iodine |
| Salt Liz is healthy, no explanation as to why given |
| Salt Liz contains potassium |
| Salt Liz is less flavorful because it contains less iodine |
| Salt Liz is less flavorful because it contains potassium |
